# Supplementary material for: From isolated polyelectrolytes to star-like assemblies: the role of sequence heterogeneity on the statistical structure of the intrinsically disordered neurofilament-low tail domain
Source: Eur Phys J E Soft Matter. 2024 Feb 15;47(2):13. doi: 10.1140/epje/s10189-024-00409-8 (PMC10869404; doi:10.1140/epje/s10189-024-00409-8)
Supplement: Supplementary file 1 — (pdf 2840 KB) [file 10189_2024_409_MOESM1_ESM.pdf]

Supplementary Information

From isolated polyelectrolyte to star-like assemblies: the role of sequence heterogeneity on the statistical structure of the intrinsically disordered Neurofilament-low tail domain

Mathar Kravikass, Gil Koren, Omar Saleh, Roy Beck
Corresponding E-mail:roy@tauex.tau.ac.il

Contents:
Table S1-S5,
Figure S1-S10

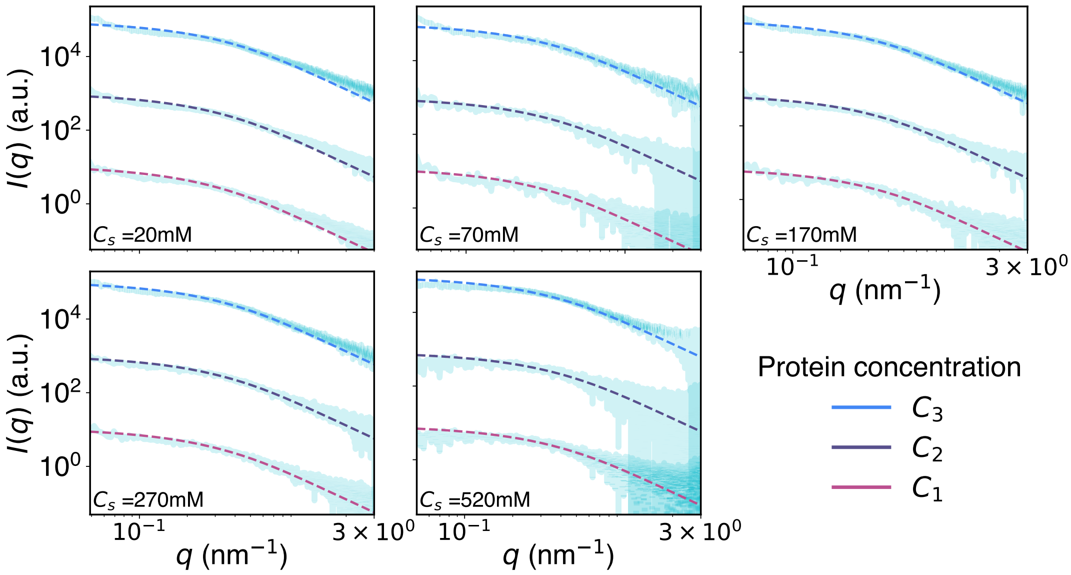

Fig. S1 ΔN42 SAXS measurements Gaussian form factor fitting for all salinity concentrations C_s. R_G used for the Gaussian form factor is as obtained by the Extended Guinier analysis (see table S1).

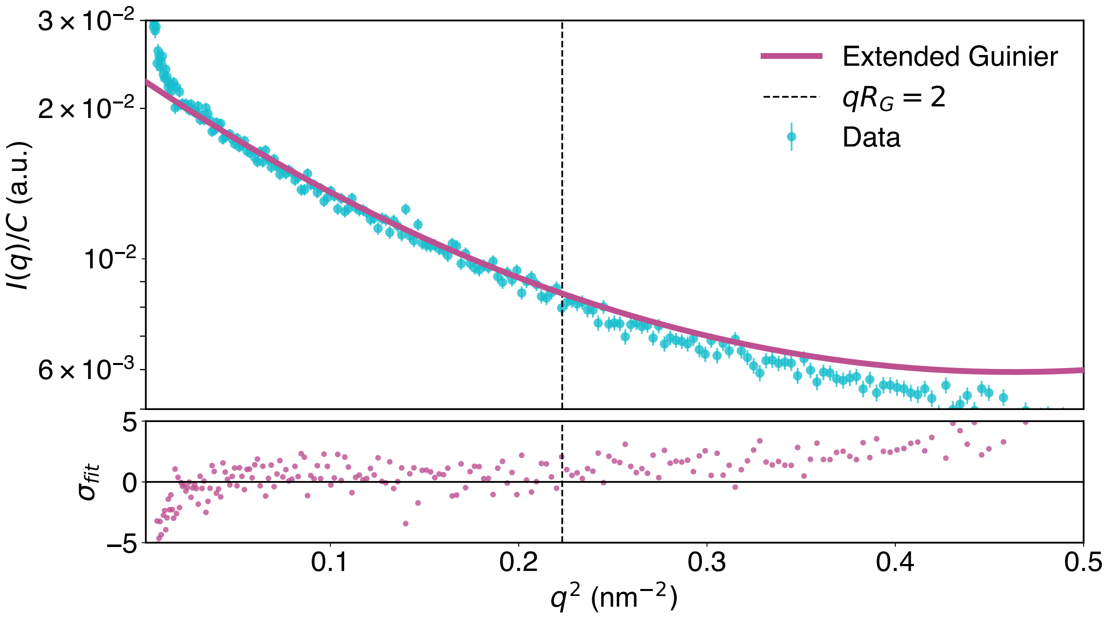

Fig. S2 ΔN42 SAXS measurement with corresponding extended Guinier curve. Bottom: Deviation from fit $\sigma_{fit} = (Y_{data} - Y_{fit})/\sigma_{data}$ Dashed line represents the maximum analysis point $qR_G = 2$ from which deviation starts. Displayed data: 20mM Tris pH8.0 in 1.1mg/ml.

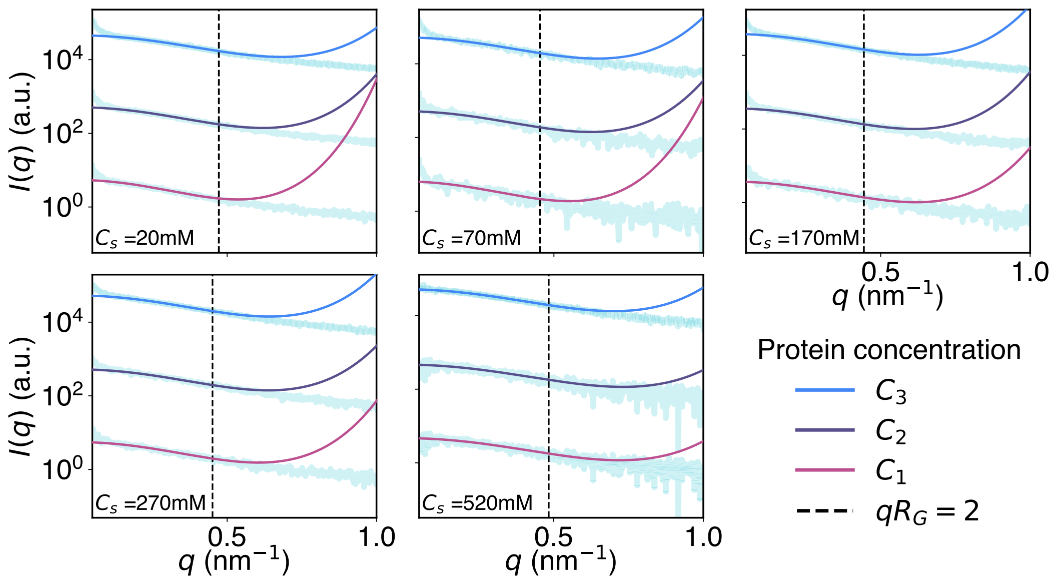

Fig. S3 ΔN42 SAXS measurements with corresponding extended Guinier curves. Dashed lines represent the maximum analysis point $qR_G = 2$ from which deviation starts. Protein concentrations were offset for clarity, with the lowest (blue) being of the highest concentration.

C_s (mM)	C (mg/ml)	R_G (nm)	ν	I_0 (cm^{-1})
20	1.1	4.23 ± 0.05	0.642 ± 0.002	0.0228
20	0.8	4.56 ± 0.07	0.660 ± 0.66	0.0254
20	0.6	5.11 ± 0.13	0.689 ± 0.007	0.027
70	1	4.41 ± 0.12	0.652 ± 0.007	0.0259
70	0.5	4.53 ± 0.22	0.659 ± 0.012	0.0257
70	0.3	4.99 ± 0.46	0.683 ± 0.023	0.032
170	1.5	4.51 ± 0.05	0.657 ± 0.003	0.19
170	0.8	4.59 ± 0.11	0.662 ± 0.006	0.18
170	0.3	4.56 ± 0.27	0.661 ± 0.015	0.18
270	1	4.43 ± 0.06	0.653 ± 0.003	0.026
270	0.5	4.45 ± 0.1	0.654 ± 0.006	0.026
270	0.3	4.64 ± 0.16	0.664 ± 0.009	0.028
520	1.5	4.14 ± 0.02	0.636 ± 0.001	0.026
520	0.78	4.00 ± 0.08	0.628 ± 0.005	0.023
520	0.38	4.05 ± 0.35	0.630 ± 0.02	0.023

Table S1 ΔN42 Extended guinier analyis data. Analysis parametres (radius of gyration R_G, scaling exponent ν, and scattering intensity at $q = 0$ (I_0)) obtained for different salt concentrations (C_s) and protein concentrations (C).

C_s (mM)	R_G (nm)	ν
20	5.76 ± 0.31	0.729 ± 0.015
70	4.84 ± 0.27	0.677 ± 0.015
170	4.71 ± 0.04	0.669 ± 0.003
270	4.61 ± 0.16	0.663 ± 0.009
520	3.88 ± 0.04	0.620 ± 0.003

Table S2 Zero concentration extended Guinier analysis data. Analysis parameters (radius of gyration R_G and scaling exponent ν) were extrapolated to zero protein concentration at various salt concentrations (C_s).

C_s (mM)	C (mg/ml)	$h/2$ (nm)	ν	Z	n	R (nm)	L (nm)	V (nm^3)	β_t (10^3nm)	β_c (10^3nm)
20	2.68	9.16±0.15	0.786±0.0033	1.60±0.03	10.11±0.711	0.89±0.028	1.19±0.09	2.26±0.027	0.227	3.826
20	1.8	8.20±0.13	0.758±0.0032	1.83±0.04	8.52±0.059	0.89	1.19	2.26	0.195	3.558
20	1	8.57±0.17	0.768±0.0041	1.87±0.05	8.12±0.060	0.89	1.19	2.26	0.195	3.558
20	0.5	8.27±0.16	0.759±0.0040	1.91±0.05	7.78±0.057	0.89	1.19	2.26	0.195	3.558
170	1.3	9.96±0.03	0.796±0.0006	3.34±0.02	2.52±0.009	0.66±0.005	X	1.18±0.007	0.039	4.014
170	0.73	10.11±0.05	0.799±0.0009	3.27±0.03	2.32±0.014	0.63±0.007	X	1.06±0.010	0.039	4.014
170	0.57	10.37±0.08	0.806±0.0015	2.13±0.02	3.10±0.037	0.60±0.006	X	0.93±0.008	0.074	3.98
170	0.24	10.78±0.10	0.815±0.0019	2.71±0.05	3.23±0.025	0.66±0.011	X	1.23±0.017	0.074	3.98
270	2	9.40±0.02	0.781±0.0003	5.37±0.02	1.89±0.007	0.70±0.004	X	1.42±0.008	0.018	4.03
270	1.5	9.43±0.02	0.782±0.0004	5.02±0.02	1.93±0.009	0.69±0.005	X	1.36±0.009	0.018	4.03
270	0.69	9.85±0.04	0.793±0.0009	4.05±0.04	2.70±0.016	0.71±0.009	X	1.53±0.016	0.039	4.014
370	2.3	9.36±0.01	0.780±0.0003	6.68±0.03	1.48±0.008	0.69±0.005	X	1.38±0.009	0.018	4.036
370	1.5	9.44±0.02	0.782±0.0003	6.43±0.03	1.65±0.009	0.71±0.006	X	1.49±0.010	0.018	4.036
370	0.96	9.53±0.02	0.785±0.0004	5.85±0.03	2.08±0.010	0.74±0.006	X	1.70±0.012	0.039	4.014
370	0.6	9.81±0.03	0.792±0.0007	5.33±0.05	2.13±0.017	0.72±0.010	X	1.59±0.019	0.039	4.014
520	2.5	9.57±0.01	0.786±0.0003	8.15±0.04	1.82±0.008	0.79±0.005	X	2.07±0.012	0.018	4.036
520	1.19	9.28±0.02	0.778±0.0004	6.66±0.04	1.57±0.010	0.70±0.006	X	1.47±0.012	0.018	4.036
520	0.45	9.82±0.03	0.792±0.0005	6.36±0.06	1.94±0.016	0.74±0.010	X	1.72±0.020	0.039	4.014

Table S3 WT spherical and cylindrical fitting analysis data. Analysis parameters (brush height (h), scaling exponent (ν), aggregation number (Z), core peptide length (n), core radius (R), cylindrical core length (L), core volume (V), tail scattering length (β_t) and core scattering length (β_c)) obtained for different salt concentrations (C_s) and protein concentrations (C). Cylinder length L values are only relevant to $C_s = 20$mM where a cylindrical core fit was used. For the cylindrical core, the same values of L and R were used for all concentrations to alleviate fitting errors (see Methods).

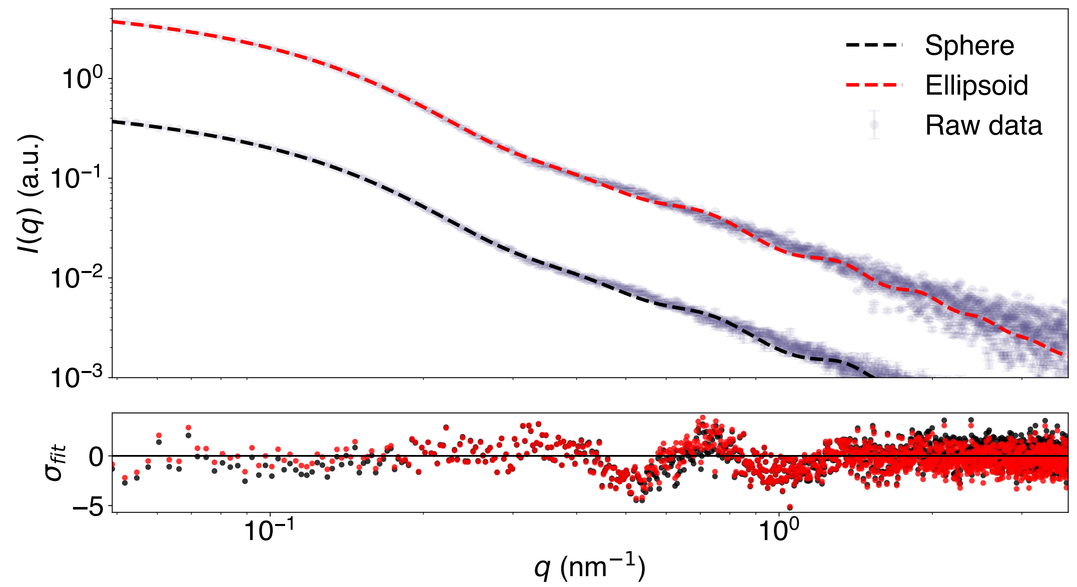

Fig. S4 SAXS measurements of WT and its fitting to different form factors. Both form factors are of the same model but use a different core: Spherical or Ellipsoidal. Spherical core fitting yields a core radius of $R = 0.66 \pm 0.016$ nm, and the ellipsoidal core yields a core radius of $R = 1.335 \pm 0.23$ nm and a secondary radius of ϵR where $\epsilon = 0.153 \pm 0.08$. Both fittings yield close values of aggregation number Z (3.046 ± 0.04 for spherical and 3.562 ± 0.07 for ellipsoidal) and tail height $h/2$ (9.838 ± 0.04 nm for spherical and 9.584 ± 0.12 for ellipsoidal). Below: Fitting error $\sigma_{fit} = (Y_{fit} - Y_{data})/\sigma_{data}$. Both curves show similar error profiles. The spherical model proved best to describe the model due to its simplicity. Displayed data: WT in 20 mM Tris pH=8.0, and 170 mM NaCl at a concentration of 1.3 mg/ml.

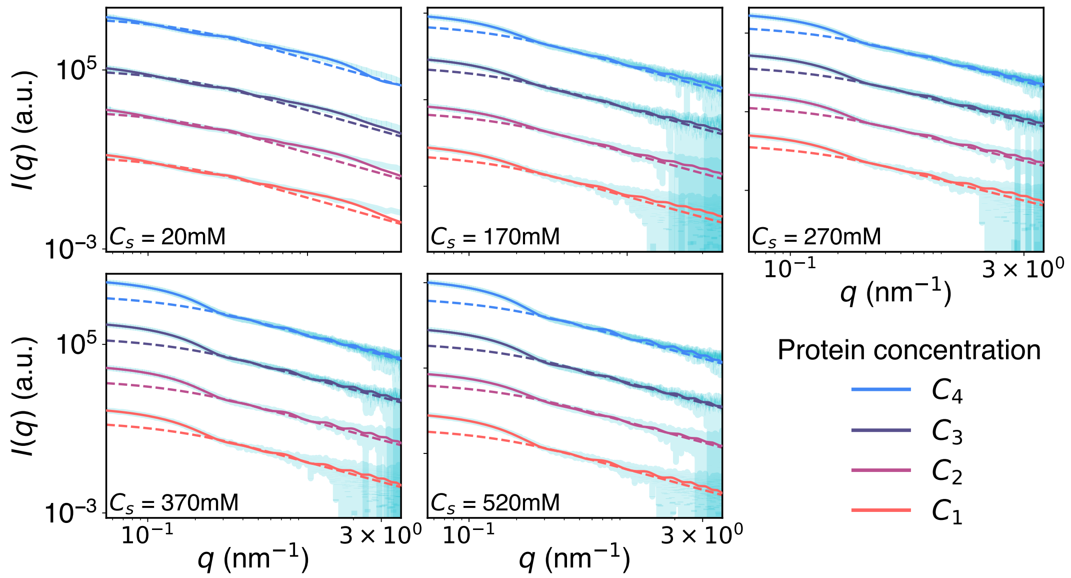

Fig. S5 SAXS measurements and spherical form factor fitting for all salinity concentrations (C_s). The $C_s = 20$ mM data fit is to a cylindrical core. Dashed lines represent the Gaussian form factor of the structure tails. Protein concentrations were offset for clarity, with the lowest (blue) being the highest concentration.

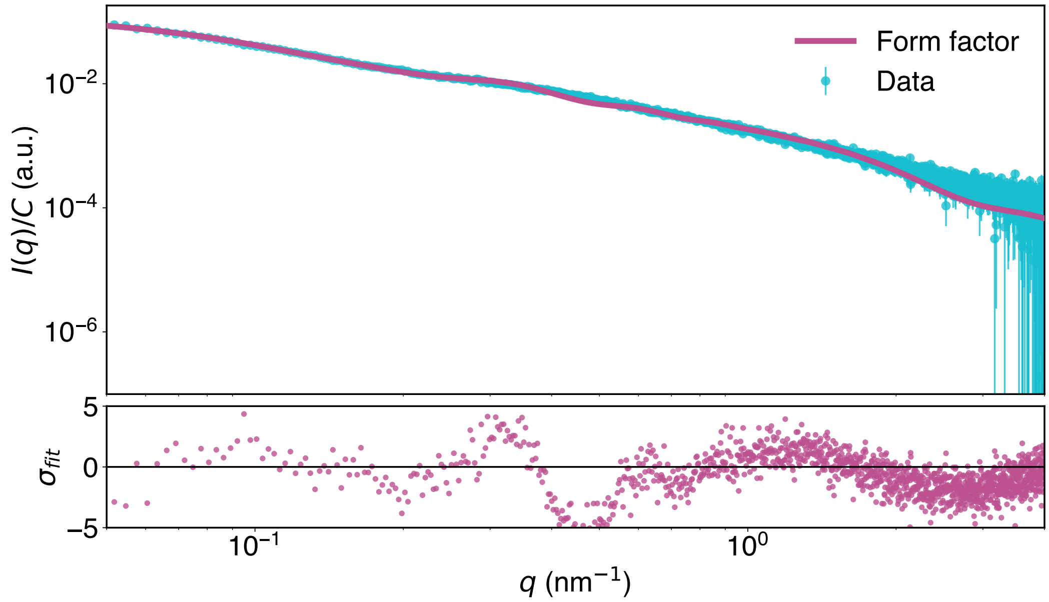

Fig. S6 WT SAXS measurement with cylindrical fitting. Measurements at the highest concentration of $C = 2.68$ mg/ml, in a 20 mM Tris buffer at pH=8.0. To alleviate fitting inconsistencies, consequent fittings of measurements with lower protein concentrations in the same buffer were done using the obtained core parameters: Core radius $R = 0.89 \pm 0.03$ nm, and core length $L = 1.19 \pm 0.09$ nm.

C_s (mM)	$h/2$ (nm)	ν	Z	n	R (nm)	L (nm)	V (nm^3)
20	8.01±0.46	0.751±0.013	2.03±0.08	7.08±0.39	0.89	1.19	2.26
170	10.60±0.33	0.811±0.008	2.83±0.31	3.17±0.55	0.64±0.03	X	1.10±0.16
270	9.84±0.21	0.793±0.006	3.52±0.22	3.06±0.30	0.70±0.03	X	1.49±0.20
370	9.73±0.12	0.790±0.003	5.19±0.29	2.39±0.12	0.76±0.02	X	1.67±0.01
520	9.47±0.44	0.783±0.011	5.67±0.35	1.82±0.29	0.68±0.06	X	1.28±0.38

Table S4 Zero concentration WT spherical and cylindrical fitting analysis data. Analysis parametres (brush height (h), scaling exponent (ν), aggregation number (Z), core peptide length (n), core radius (R), cylindrical core length (L) and core volume (V)) were extrapolated to zero protein concentration at various salt concentrations (C_s). Cylinder length L values are only relevant to $C_s = 20$mM where a cylindrical core was used.

C_s (mM)	A_2^{WT} (cm^{3}mol/g^2 $\times$ 10^3)	$A_2^{\Delta N42}$ (cm^{3}mol/g^2 $\times$ 10^3)
20	-0.295±1.346	13.264±0.466
70	X	3.978±1.248
170	-2.072±2.091	0.169±1.544
270	-3.328±0.508	-1.152±0.756
370	-2.020±0.563	X
520	-1.933±3.582	-4.417±1.514

Table S5 Second virial coefficient A_2 values for both variants in salt concentration C_s.

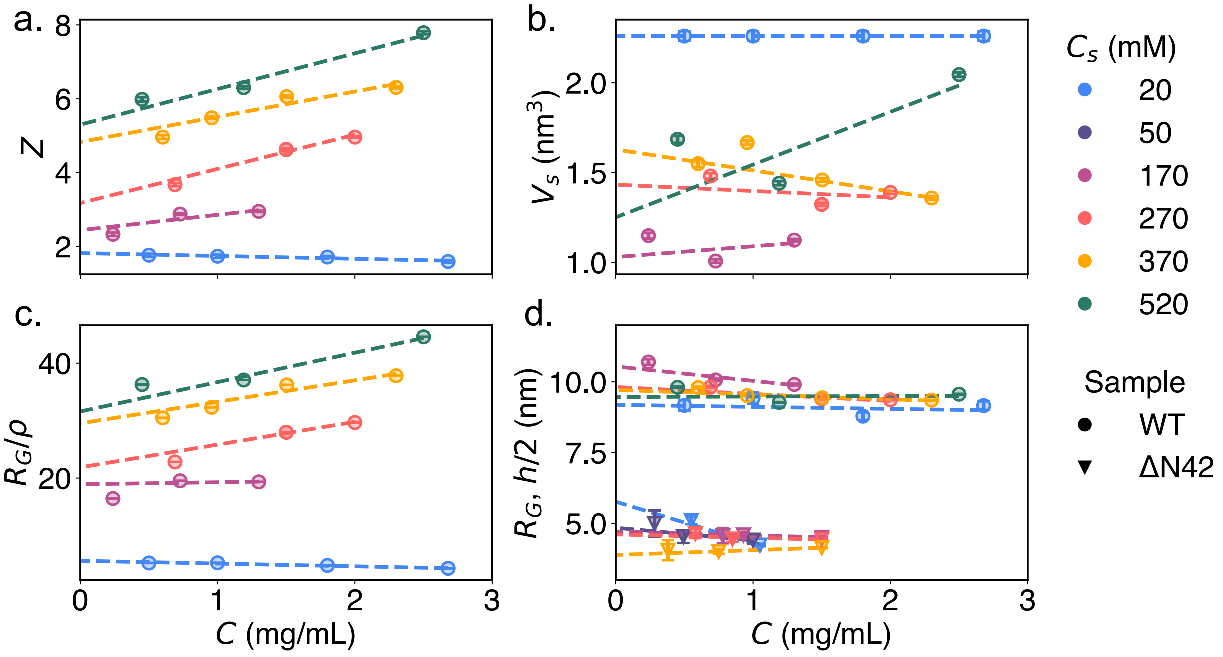

Fig. S7 Structural parameters for WT (circles) and ΔN42 (triangles) variants extracted from fitting the SAXS data. Dashed lines demonstrate the linear fitting of the data used to obtained the zero concentration extrapolations. **a.** Aggregation number (Z) dependency on protein concentration (C) increases with increasing salt. **b.** Core volume V_s against protein concentration (C). In $C_s = 20$ mM, the V_s values are constant due to fitting constraints (see Methods). **c.** In all cases, the tail heights (h) are much larger than the corresponding grafting length (ρ), indicative of a brush regime. **d.** The structurally intrinsically disordered ΔN42 variant compacts with higher C_s values and remains more compacted from the projected tails for the WT variant. For the ΔN42 variant R_G drastically changes as a function of the protein concentration (C).

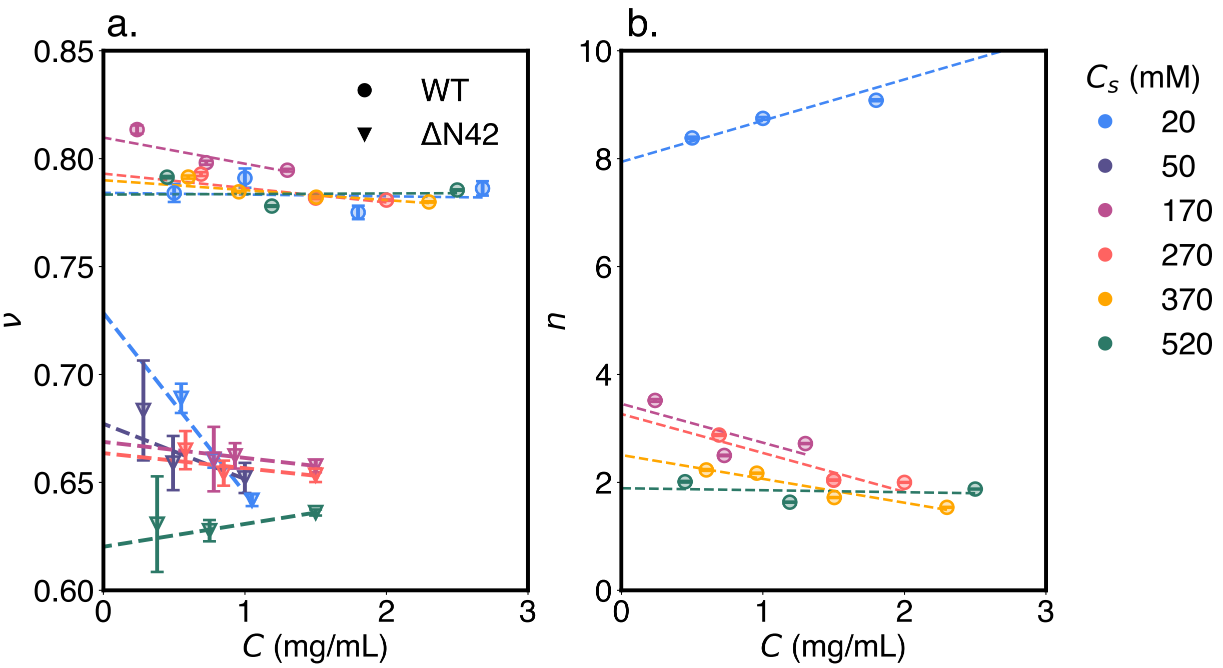

Fig. S8 **a.** Flory exponent (ν) of WT tails and ΔN42 variants as a function of the concentration. ΔN42 shows to change radically as a function of the concentration at the lowest salinities. This effect is reduced as salinity concentration Cs reaches 170mM. WT and the rest of ΔN42 ν data shows little change as a function of the protein concentration.**b.** The core (aggregated) peptide length per polypeptide as a function of the concentrations. The large drop observed from C_s = 20mM to C_s = 170mM can be attributed to the shift from a dimer to a trimer. Core peptide length difference diminishes with increasing salinity, however the value still remain largely similar.

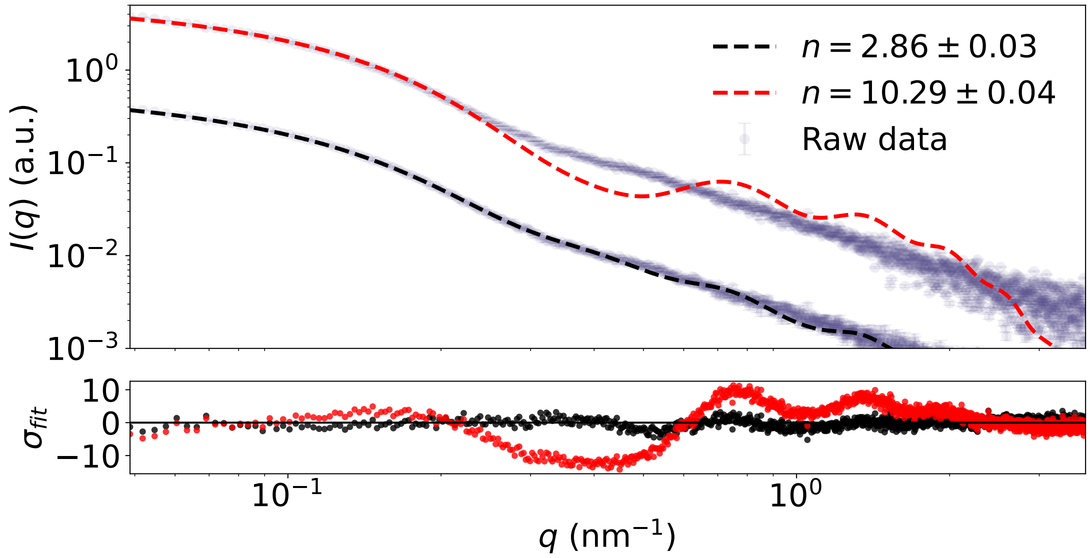

Fig. S9 SAXS measurement of WT and its fitting with different core residue number n. In fixing the core residue number to a constant value of 10 (in red), the fitting becomes noticeably worse than when n is allowed to vary (in black). Displayed data: WT in 20 mM Tris pH=8.0, and 170 mM NaCl at a concentration of 1.3 mg/ml.

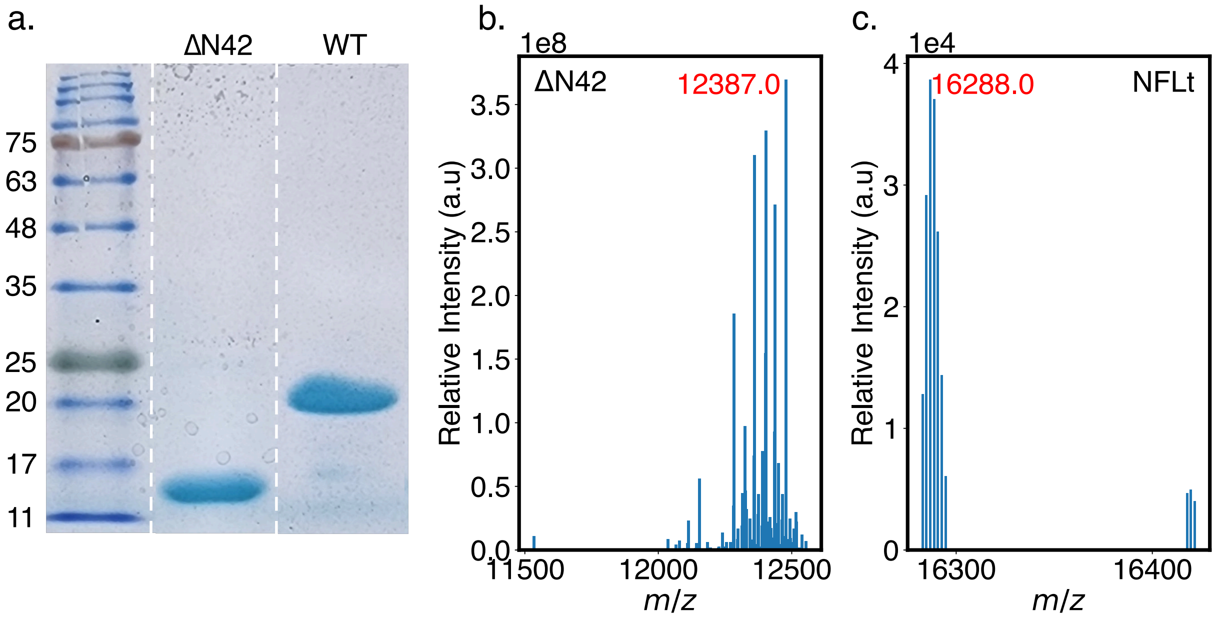

Fig. S10 **a.** SDS-PAGE Tris-Glycine 15% of both ΔN42 and NFLt (WT), showing purity above 95%. White dashed lines indicate where image lanes were edited closer for clarity. Both show a higher molecular weight reading in the gel, which is common for IDPs.**b-c.** Deconvoluted ESI-TOF MS spectra of ΔN42 and NFLt respectively. Theoretical molecular weight values are 12423.57 and 16233.79 for ΔN42 and NFLt, respectively
